# Supplementary material for: Microscale carbon distribution around pores and particulate organic matter varies with soil moisture regime
Source: Nat Commun. 2022 Apr 21;13:2098. doi: 10.1038/s41467-022-29605-w (PMC9023478; doi:10.1038/s41467-022-29605-w)
Supplement: Supplementary file 1 — Supplementary Information [file 41467_2022_29605_MOESM1_ESM.pdf]

Supplementary material

Microscale carbon distribution around pores and particulate organic matter varies with soil moisture regime

Correspondence: steffen.schlueter@ufz.de

Supplementary Table 1: Summary of site description, bulk properties, organic matter fractions in soil aggregates and microscale properties based on imaging for three investigated soils. Values in brackets represent standard deviations.

	property (unit)	conditions and remarks	Haplic Chernozem	Fluvial Gleysol	Stagnic Luvisol
site	P (mm)		484	600	856
	T (°C)		8.8	9.3	8.2
	elevation (m a.s.l)		116	172	360
	coordinates		N51°23', E11°52'	N50°32', E8°43'	N48°21', E13°11'
	land use	(duration of current use in years)	cropland (>100)	grassland (>100)	cropland (>30)
	plant species	crop rotation or composition	maize, barley, maize, wheat	>50% grasses, >40% forbs, <2% legumes	wheat, maize, wheat, triticale
	tillage		conventional	none	conventional
	soil depth (cm)		0–30	5–20	5–20
bulk soil	clay content (g g^{-1} %)		21	27	16
	silt content (g g^{-1} %)		68	41	73
	sand content (g g^{-1} %)		11	32	11
	clay mineralogy		Illite > Kaolinite > Vermiculite	Vermiculite > Illite > chlorite = Kaolinite	Illite > Kaolinite > Vermiculite
	pH	in $CaCl_2$	6.6	5.7	6.7
	cation exchange capacity ($mmol_c$ kg^{-1} soil)	potential CEC at pH 7 or 8.1	294	321	150
	Fe_d (g kg^{-1} soil)	dithionite-extractable fraction	5.9	4.1	11.8
	Fe_o (g kg^{-1} soil)	oxalate-extractable fraction	1.2	7.1	5.8
	Al_o (g kg^{-1} soil)	oxalate-extractable fraction	1.2	1.3	1.1
	POM total (mg C g^{-1} soil)	density fractionation (<1.6 g cm^{-3})	5.2 (1.3)	3.3 (0.4)	2.4 (0.3)
	POM free (mg C g^{-1} soil)	without ultrasonication	2.4 (1.2)	2.1 (0.2)	0.8 (0.0)
	POM occluded (mg C g^{-1} soil)	with ultrasonication	2.8 (0.5)	1.2 (0.3)	1.6 (0.3)
	MAOM (mg C g^{-1} soil)	density fractionation (>1.6 g cm^{-3})	19.3 (0.3)	38.3 (1.6)	10.4 (0.1)
	WEOC (µg C g^{-1} POM)	soil:water 1:50	4.21 (0.16)	4.71 (0.23)	3.01 (0.59)
aggregate (d: 2–8 mm)	TOC (mg g^{-1} soil)		22.1 (0.3)	39.5 (3.9)	12.0 (0.1)
	TN (mg g^{-1} soil)		1.8 (0.1)	4.0 (0.2)	1.3 (0.0)
	WEOC (µg g^{-1} soil)	soil:water 1:5	158 (14)	445 (33)	155 (14)
	CO_2 efflux (µg C g^{-1} soil day)	0% O_2, 60% WFPS, mixed with silt	4.5 (0.2)	21.3 (1.1)	7.2 (0.3)
	CO_2 efflux (µg C g^{-1} soil day)	20% O_2, 100% WFPS	16.8 (1.2)	92.1 (16.1)	18.9 (2.5)
	CO_2 efflux (µg C g^{-1} soil day)	20% O_2, 50% WFPS	12.2 (0.7)	53.8 (3.7)	16.8 (1.8)
	aggregate volume (mm^3)		184 (48)	176 (34)	156 (31)
	aggregate density (g cm^{-3})	d: 6–8 mm	1.83 (0.05)	1.76 (0.09)	1.89 (0.06)
microscale	visible porosity (mm^3 mm^{-3} %)	$V>1.8 \times 10^3$ µm³	7.5 (2.5)	11.4 (2.9)	3.3 (1.1)
	internal pore surface area density (mm^2 mm^{-3})	$V>1.8 \times 10^3$ µm³	18.1 (1.1)	18.2 (0.2)	13.7 (1.9)
	soil matrix fraction (mm^3 mm^{-3} %)	$V>1.8 \times 10^3$ µm³	89.2 (2.6)	77.2 (4.3)	91.8 (2.1)
	sand & concretions fraction (mm^3 mm^{-3} %)	$V>1.8 \times 10^3$ µm³	2.8 (0.5)	10.9 (2.2)	4.4 (0.9)
	POM fraction (mm^3 mm^{-3} %)	$V>1.8 \times 10^3$ µm³	0.7 (0.4)	0.6 (0.4)	0.5 (0.4)
	POM abundance (mm^{-3})	$V>6.3 \times 10^5$ µm³	1.33 (0.69)	1.04 (0.74)	0.76 (0.35)
	rounded POM fraction (mm^3 mm^{-3} %)	$V>6.3 \times 10^5$ µm³	0.15 (0.08)	0.13 (0.15)	0.04 (0.02)
	mean volume of rounded POM (x 10^5 µm³)	$V>6.3 \times 10^5$ µm³	143 (10)	170 (21)	131 (24)
	fibrous POM fraction (mm^3 mm^{-3} %)	$V>6.3 \times 10^5$ µm³	0.16 (0.09)	0.28 (0.42)	0.14 (0.08)
	mean volume of fibrous POM (x 10^5 µm³)	$V>6.3 \times 10^5$ µm³	149 (15)	155 (19)	150 (17)
	matrix volume fraction affected by Os gradient around pores (mm^3 mm^{-3} %)	pore distance<20–90 µm	65 (17)	74 (12)	19 (16)
	mean pore distance in matrix (µm)	$V>1.8 \times 10^3$ µm³	49 (9)	52 (19)	106 (18)
	matrix volume fraction affected by Os gradient around POM (mm^3 mm^{-3} %)	POM distance<10–80 µm	1.1 (0.2)	2.2 (0.8)	1.3 (0.4)

Supplementary Table 2: Morphological traits of POM objects used for random forest classification into compact and fibrous POM. Traits are sorted according to importance for feature detection.

trait	definition	remark
Blobness	$R_3/\sqrt{R_2 R_1}$	Objects are described as equivalent inertia ellipsoids with radii $R_1 > R_2 > R_3$. The larger R_3 in comparison to the other, the more blob-like (less elongated) the object is.
Compactness	$V/(\frac{4}{3}\pi R_1 R_2 R_3)$	The more compact, i.e. more bulky with less holes, the more similar the real volume V is to the volume of an equivalent ellipsoid
Plateness	$\lvert R_2\rvert/\lvert R_1\rvert$	In plate-like objects R_2 is much shorter than R_1.(and more similar to R_3).
Os_{CT}	$\overline{I_a} - \overline{I_b}$	Mean difference intensity in an object between image acquired before Os staining $\overline{I_b}$ and after Os staining $\overline{I_a}$

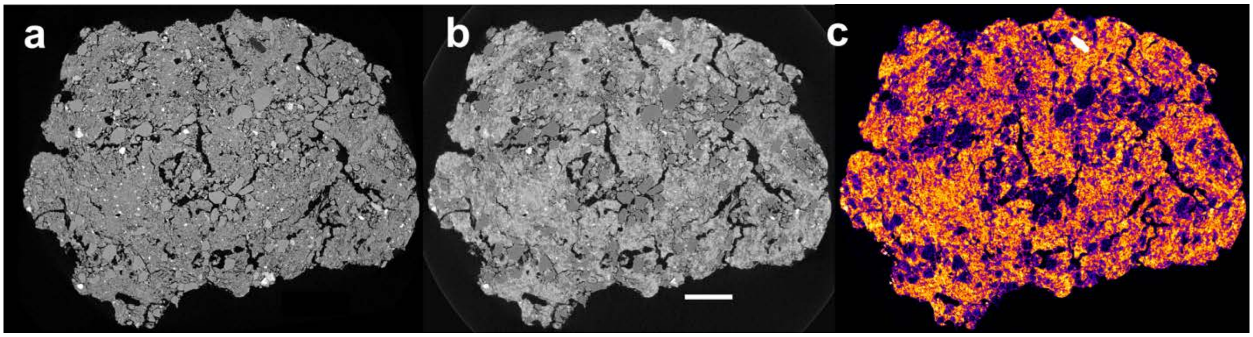

Supplementary Figure 1: Two-dimensional slices of registered, gray-value normalized X-ray tomograms acquired (a) before and (b) after Os staining and the associated difference image in pseudo colors. Scale bar represents 1 mm.

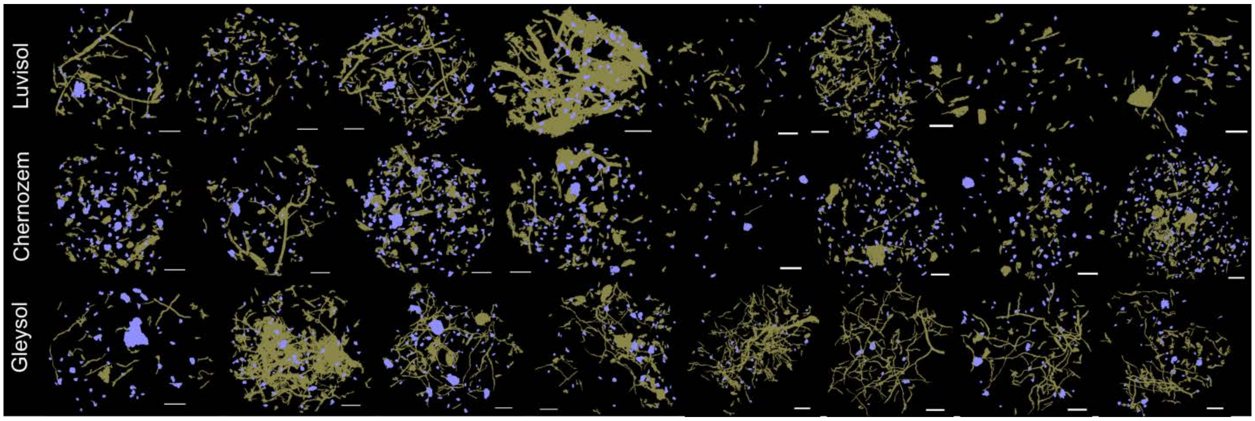

Supplementary Figure 2: Two-dimensional projections through complete aggregates displaying fibrous and rounded POM (>6.3 × 10^5 µm³ each) in gold and purple, respectively. Scale bars represent 1 mm.

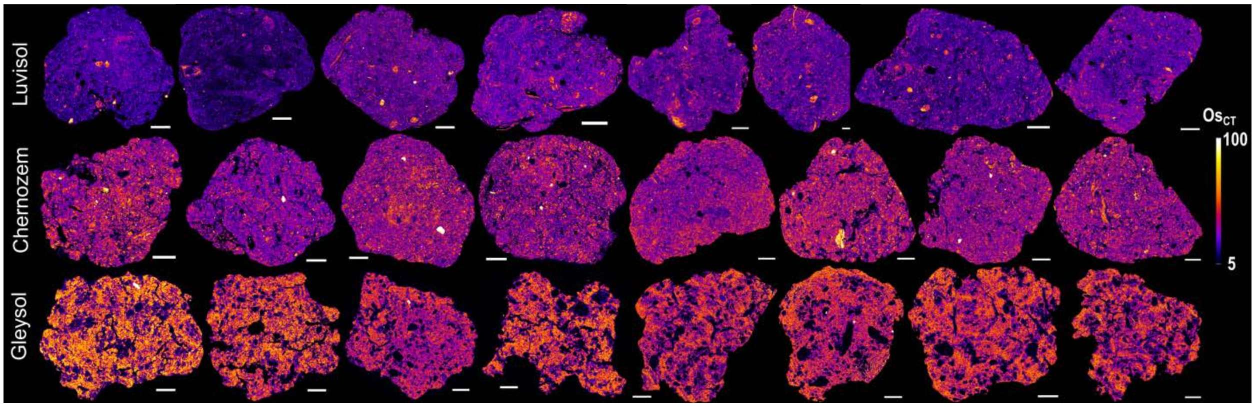

Supplementary Figure 3: Representative two-dimensional slices of difference image reflecting local Os intensity in each investigated soil aggregate. Scale bars represent 1 mm.

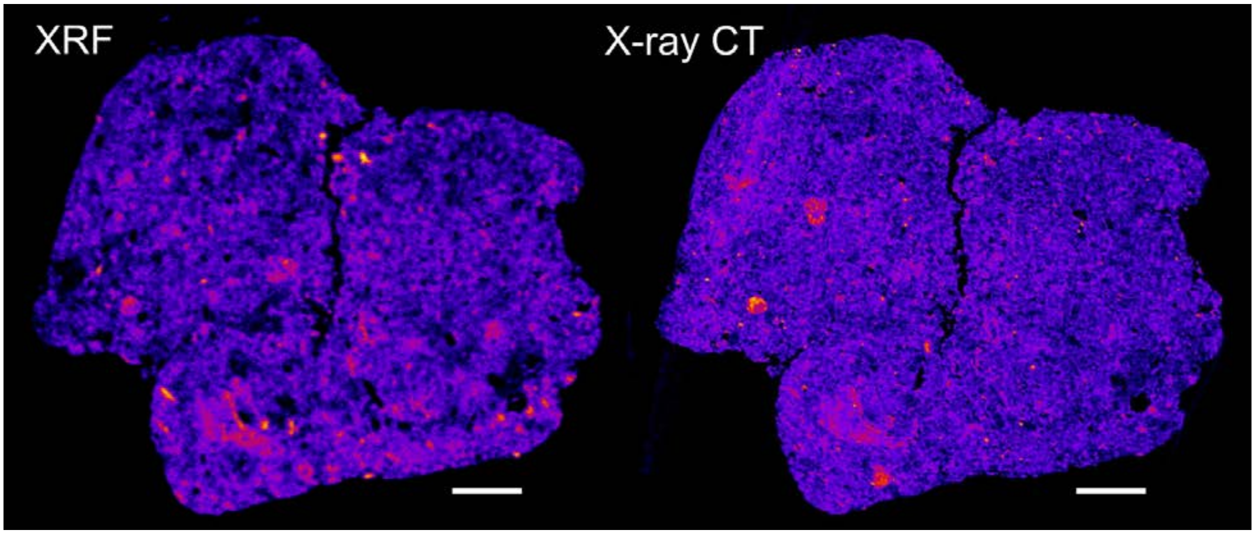

Supplementary Figure 4: The comparison of spatial Os distribution in a soil section mapped with µXRF (lateral resolution 20 µm, thickness 80 µm) and the registered X-ray CT plane (5 µm isotropic resolution) indicates general congruency with small differences mainly due to different depth resolution. Scale bar represents 1 mm.

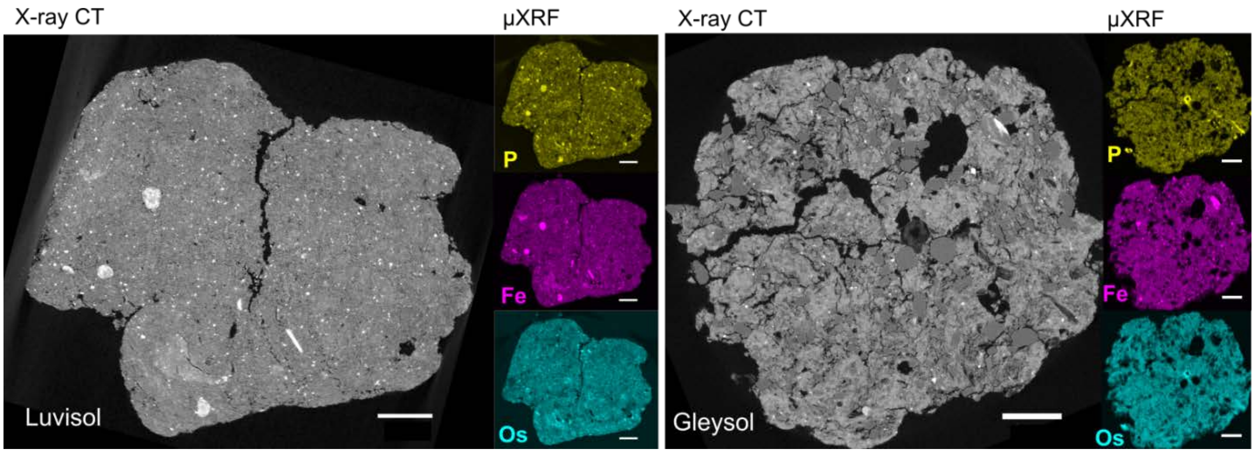

Supplementary Figure 5: Two-dimensional cross sections through a Stagnic Luvisol and Fluvic Gleysol aggregate scanned with X-ray CT after Os staining. The cross sections correspond to the exposed surfaces of a soil sections that are mapped with µXRF. In addition to Os, P is shown as a proxy for organic molecules and Fe as an indicator of Fe-rich nodules in hydromorphic soils.

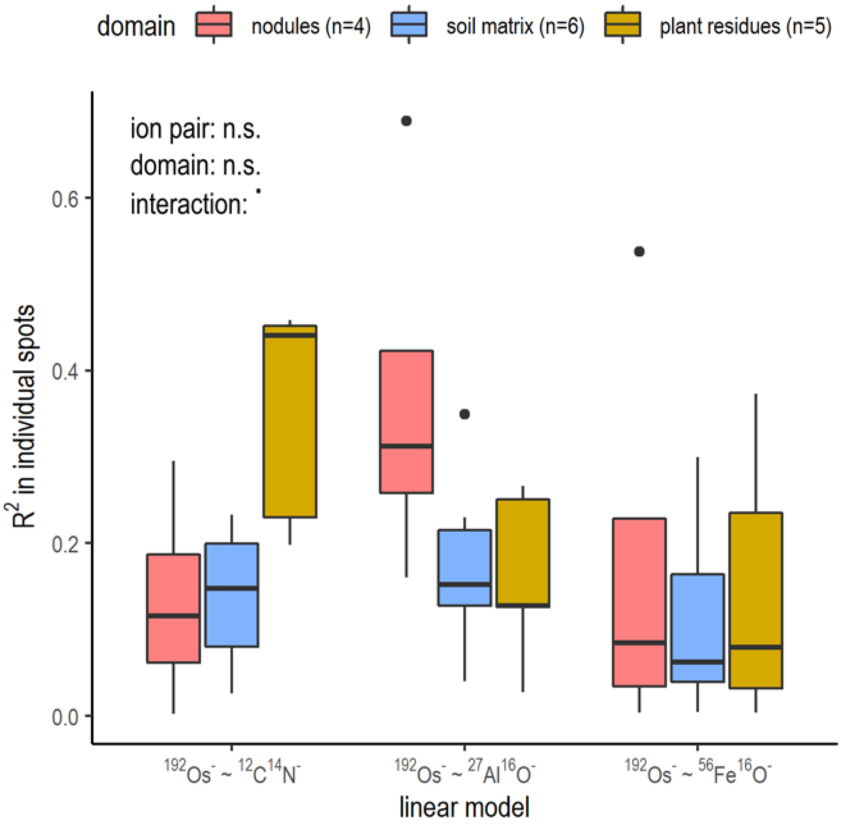

Supplementary Figure 6: Explained variability (R^2) of ^{192}Os intensity by intensity of other ions ($^{12}C^{14}N$, ^{27}Al, ^{56}Fe) in selected spots from different domains mapped with NanoSIMS. In each spot co-localized ion intensities are mapped at a downscaled resolution of 1 µm resulting in one R^2 value per spot and ion pair. Boxplots represent

the 0%, 25%, 50%, 75% and 100% percentiles after outlier detection for several spots (n = 4–6) of each domain. Two-way ANOVA was carried out with the factors ion pair and domain.

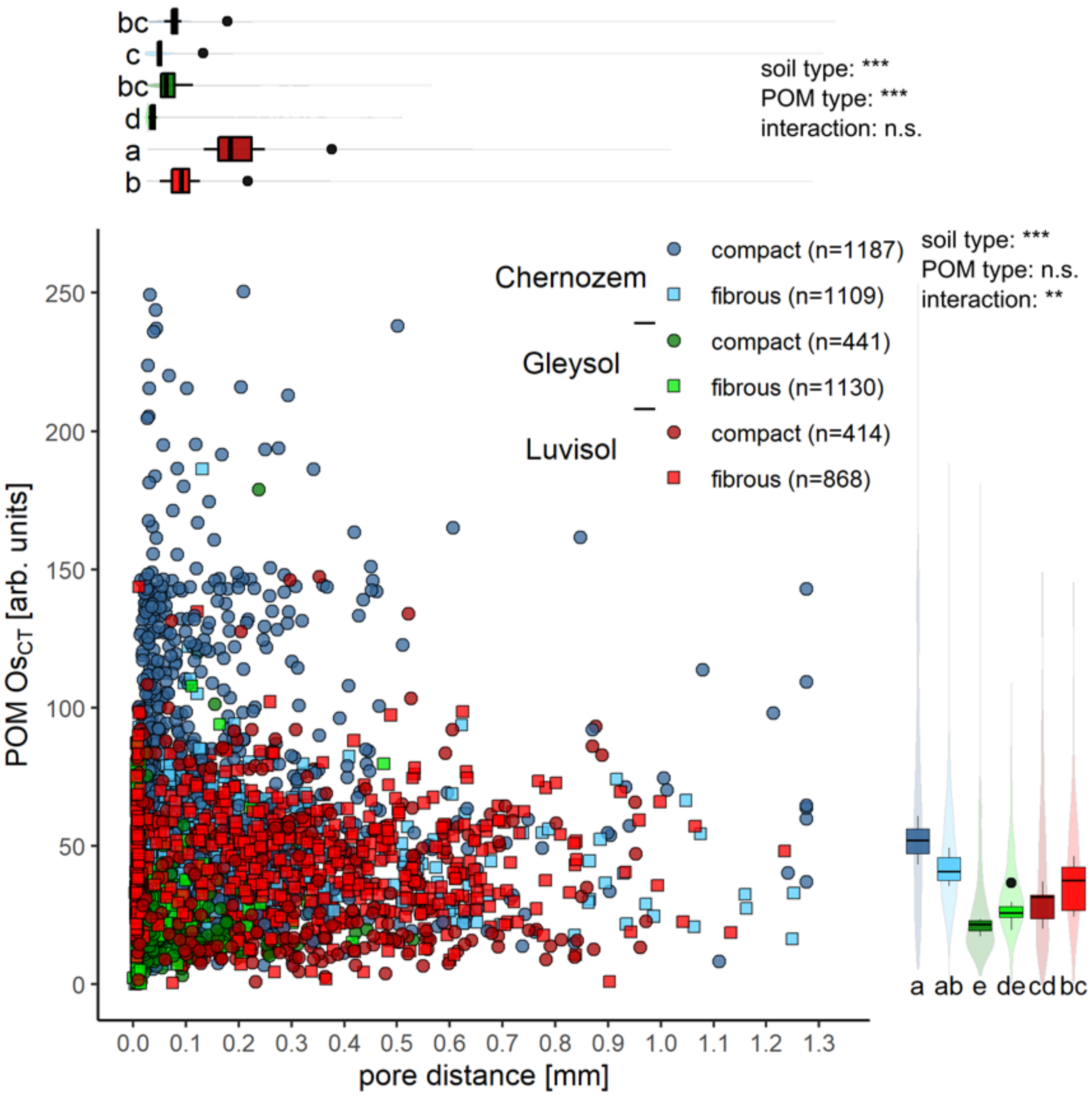

Supplementary Figure 7: Average Os concentration and average pore distance for each individual POM particle, which has been segmented into fibrous and compact POM by a random forest classifier. Pore distances are computed after excluding pore objects that are disconnected from the aggregate boundary irrespective of size. This is a situation more likely to be encountered for distances to air-filled pores, when a soil is drained from full saturation to field capacity. Violin plots represent marginal distributions of individual POM objects. Boxplots represent the 0%, 25%, 50%, 75% and 100% percentiles of averages for individual aggregates (n=8) with small letters reflecting significant differences tested at $p < 0.05$ (n.s. not significant).

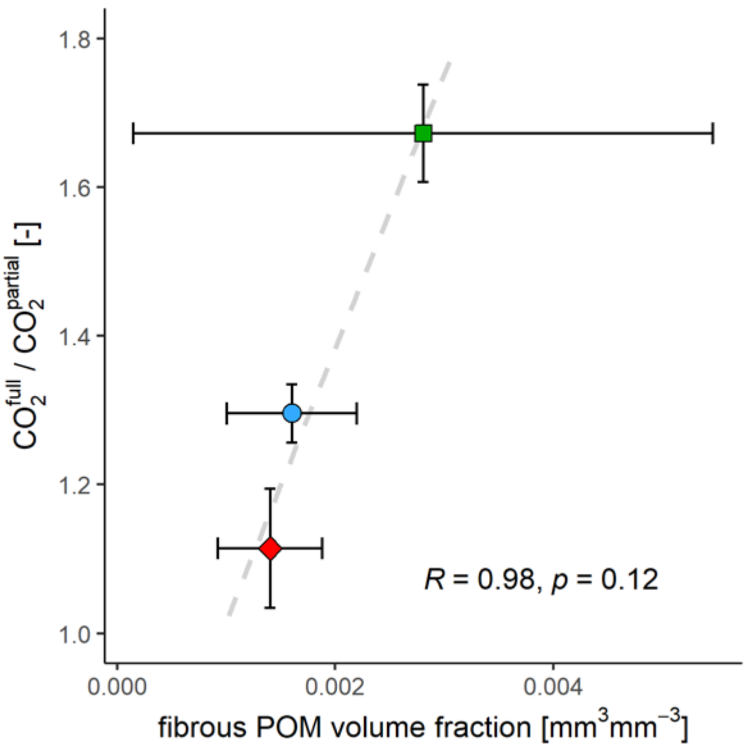

Supplementary Figure 8: Ratio between CO_2 efflux at fully (100% WFPS) and partially water-saturated (50% WFPS), oxic incubation of soil aggregates as a function of the image-derived volume fraction of particulate organic matter (POM) with elongated morphology representing fibrous POM mainly residing in the intact pore network. Bars represent two standard errors in each direction with n = 3 for CO_2 data and n = 8 for image-derived POM data.

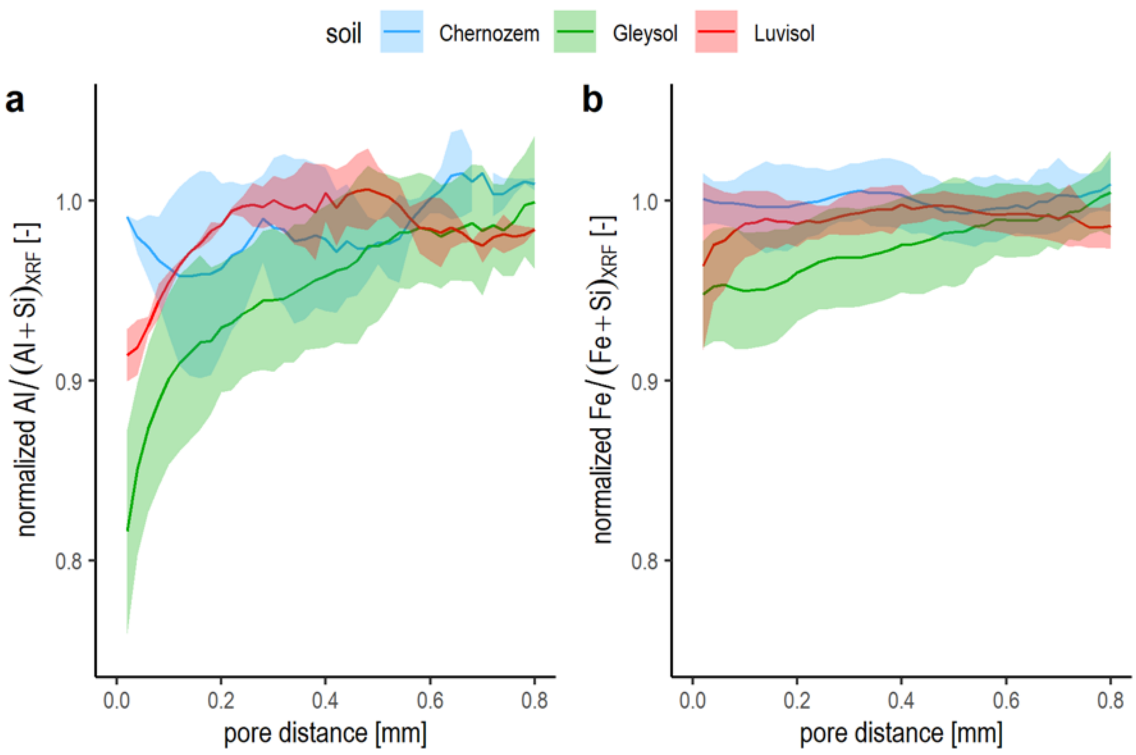

Supplementary Figure 9: (a) Average Al and (b) average Fe intensity each normalized by co-located Si intensity in elemental maps of soil sections obtained with µXRF (11−14 sections for each soil). Normalized intensities are shown as a function of pore distance in the soil matrix. Note that spatial resolution (20 µm lateral, 40−400µm depth resolution depending on section thickness) and thus the smallest visible pores are much coarser in µXRF as compared to X-ray CT (isotropic 5 µm).
